# Short-term effects of ambient particulates and gaseous pollutants on the incidence of transient ischaemic attack and minor stroke: a case-crossover study

**DOI:** 10.1186/1476-069X-11-77

**Published:** 2012-10-15

**Authors:** Getahun Bero Bedada, Craig J Smith, Pippa J Tyrrell, Adrian A Hirst, Raymond Agius

**Affiliations:** 1 Institute of Environmental Medicine, Unit of Environmental Health, Karolinska Institutet, Scheele lab, 5th floor, Nobels väg 13, Solna Campus, SE-171 77, Stockholm, Sweden; 2 Centre for Epidemiology, Institute of Population Health, The University of Manchester, Ellen Wilkinson Building, Oxford Road, Manchester, M13 9PL, UK; 3 Brain Injury Research Group, School of Biomedicine, The University of Manchester, Clinical Sciences Building, Manchester Academic Health Science Centre, Salford Royal Foundation Trust, Manchester, M6 8HD, UK

**Keywords:** Particulate and gaseous air pollution, Transient ischaemic attack, Minor stroke

## Abstract

**Background:**

While several studies have investigated the effects of short-term air pollution on cardiovascular disease, less is known about its effects on cerebrovascular disease, including stroke and transient ischaemic attack (TIA). The aim of the study was to assess the effects of short-term variation in air pollutants on the onset of TIA and minor stroke.

**Methods:**

We performed secondary analyses of data collected prospectively in the North West of England in a multi-centre study (NORTHSTAR) of patients with recent TIA or minor stroke. A case - crossover study was conducted to determine the association between occurrence of TIA and the concentration of ambient PM_10_ or gaseous pollutants.

**Results:**

A total of 709 cases were recruited from the Manchester (n = 335) and Liverpool (n = 374) areas. Data for the Manchester cohort showed an association between ambient nitric oxide (NO) and risk of occurrence of TIA and minor stroke with a lag of 3 days (odds ratio 1.06, 95% CI: 1.01 – 1.11), whereas negative association was found for the patients from Liverpool. Effects of similar magnitude, although not statistically significant, were generally observed with other pollutants. In a two pollutant model the effect of NO remained stronger and statistically significant when analysed in combination with CO or SO_2_, but was marginal in combination with NO_2_ or ozone and non-significant with PM_10_. There was evidence of effect modification by age, gender and season.

**Conclusions:**

Our data suggest an association between NO and occurrence of TIA and minor stroke in Greater Manchester.

## Background

Epidemiological studies have consistently shown associations between increased ambient air pollution and increased cardio-pulmonary mortality, morbidity and emergency department visits [1]. Admission rates and emergency department visits for stroke are increased following short-term variations in ambient particulate and gaseous pollution [2,3] and few studies have reported effects of short term changes in air pollution on TIA and stroke [4-6].

The biological mechanisms underlying the link between air pollution and stroke are unclear. Cerebrovascular risks of air pollution may be related to increased coagulability of blood [7,8], destabilization of atheromatous plaques [9] or release of inflammatory mediators [10], all of which are associated with particulate air pollution [1].

An association between ambient air pollutants with stroke morbidity and mortality has been supported by some studies [11-15] but not others [16-18]. These discrepancies are probably accounted for by differences between populations studied (including different levels of air pollution) and methods of case ascertainment. Literature is scant on the effect of air pollution on the development of TIA and minor stroke.

Air pollution is a modifiable risk factor and understanding the risks attributed to it would enable preventive health measures to be taken. In this study, we used an existing data set, the North West of England Transient Ischaemic Attack and Minor Stroke (NORTHSTAR) study of patients with TIA or minor stroke in the North West of England. Our hypothesis is that short-term changes in ambient air pollution are related to onset of TIA and minor stroke. The primary objective of the study is to investigate the effects on the onset of TIA and minor stroke of short-term exposure to PM_10_ and the following gases: NO, NO_2,_ CO, SO_2_ and ozone.

## Methods

### Study population

The NORTHSTAR study has previously been described in detail [19]. Briefly, 709 patients with incident TIA or minor stroke were prospectively recruited from TIA services in North West England between 2003 and 2007. These patients attended one of five centres: Salford, Central Manchester and Trafford in Greater Manchester, and Walton and Aintree Hospitals in Liverpool. This multicentre study was designed to investigate the prognostic role of peripheral and genetic inflammatory markers in patients with recent index TIA and minor stroke [19,20].

TIA was defined as a clinical syndrome of focal loss of cerebral, or monocular function with resolution of symptoms within 24 h [21]. Minor stroke was defined by the same clinical syndrome, but with symptoms lasting greater than 24 h, and only minimal residual functional impairment [22]. Patients were recruited either from TIA clinics, presentation to emergency room or following admission to stroke units. Participant inclusion criteria included diagnosis of TIA and minor stroke confirmed by a consultant stroke physician or neurologist, symptom onset within the preceding 6 weeks, age >18 years without significant co-morbidity or disability. Baseline demographic data, including home postal address, vascular risk factors, medications, smoking status and date and time of qualifying index event were recorded at study entry. Ethical approval for the original project was obtained from each local ethics committee and approval for the current study was obtained from the Central Manchester Research Ethics Committee and Salford Royal NHS Foundation Trust.

### Environmental data

Environmental air quality data were obtained from the UK National Air Quality Archive available at http://www.airquality.co.uk/index.php. Hourly concentrations data on the following pollutants were acquired from 8 central monitoring stations in Greater Manchester and Liverpool: PM_10_, NO, NO_2_, CO, SO_2_ and ozone. The patients participating in this study were from five different centres clustered into two main groups: Manchester and Liverpool. As these two regions are geographically distinct, separate exposure estimates were made for each region.

Data on meteorological confounders (temperature and relative humidity) were obtained from the UK Meteorological Office database [23]. Data for Greater Manchester were obtained from the Ringway station, situated 17 km south of Manchester city centre. For Liverpool, data were obtained from the Crosby station, 13 km north of Liverpool town centre. Data on weekly influenza epidemics in the region were provided by the regional Health Protection Agency (http://www.hpa-nw.org.uk/index.htm).

From the hourly data, a 24-h average exposure parameter was created, except for ozone, for which a daytime 8-hour average was used. Four exposure lags were evaluated for the environmental data, comprising data for the same day as the TIA or minor stroke (lag 0) and the three immediately preceding days (lag 3 to lag 1).

Missing data for some days were imputed by multiple imputation, by incorporating data from other pollution monitors as well as meteorological variables. The proportion of missing values ranged from 2.3% to 49% for air pollutants and less than 1% for meteorological parameters. Where more than 25% were missing for a particular pollutant from a particular monitor, this was excluded from the imputation.

### Statistical analysis

The analysis was conducted using a case-crossover design [24] with a time stratified referent selection approach. The case-crossover design is a variant of the matched case control study and consists of only cases, which serve as their own controls in the analysis. Exposure data on the day of the health event were studied in relation to the index and compared with selected referent days. Case-crossover design inherently controls for fixed individual characteristics like gender and race, and allows the evaluation of effect modification by individual characteristics. In addition, the design controls for seasonal confounding and time varying factors [25]. A variety of referent selection schemes have been used in case-crossover design, such as unidirectional, symmetric bidirectional, full-stratum bidirectional and time stratified sampling [25-27]. Janes et al. suggest using the time stratified approach as a standard method in selecting referent periods, as it ensures unbiased estimation and avoids bias resulting from time trends in the exposure [25].

For reported date of onset of each TIA or minor stroke event, three to four control periods were selected in the same calendar month. The exposure levels of each case of TIA and minor stroke were compared with exposure on referent periods. For instance, if an individual had TIA or minor stroke on the 16^th^ July, days 2^nd^, 9^th^, 23^rd^ and 30^th^ July constituted a control set of four weekdays. In time stratified case-crossover design, the control periods for a given subject are restricted to the same weekday, month and year, which controls for season, long-term trends and day of the week [25].

To explore the relationship between each ambient air pollutant and TIA or minor stroke, conditional logistic regression analysis was performed. Adjusted odds ratios (OR) were estimated, each scaled to the IQR (inter quartile range) increase of each pollutant. The statistical model was adjusted for meteorological variables (temperature and relative humidity), UK public holidays and influenza epidemics. We first constructed a baseline model by incorporating these confounder variables without the air pollutants. We used a natural cubic spline with three degrees of freedom to model the nonlinear effect of temperature and relative humidity. We used the Akaike Information Criterion to determine the goodness of model fit and choose relevant predictors that should be included in the final model.

In a further analysis, the effect of two pollutants was explored to evaluate the potential confounding effect of co-pollutants and to identify which of the pollutants remained stable in explaining the relationship with the outcome. This was achieved by first analysing the independent effect of each pollutant and then modelling the effects of two pollutants together. Effect modification by age (cut-off at 65 years), gender and season (October to March vs. April to September) were assessed.

In a sensitivity analysis we employed a bi-directional case-crossover referent selection scheme [25,26] and evaluated exposure of cases 7 and 14 days before and after the onset of TIA.

Effect estimates were provided with 95% CI and statistical significance of p < 0.05 was used. The statistical analysis was carried out using Stata version 10.0 (Stata Corp, College Station, Texas).

## Results

### Characteristics of the study population and exposure

The analysis was based on 709 patients with TIA or minor stroke, 374 (52.8%) from Liverpool and 335 from Manchester. Baseline participant characteristics are shown in Table 1. The participants were mostly elderly patients with an age range of 27-93 years and a mean of 66.8 ± 11. About 62% of the participants were above the age of 65. Their body mass index (BMI) ranged from 14.8-55 kg/m^3^. The majority of the participants (99%) were white and they were predominantly male. One third were current smokers. The majority were prescribed medications such as antihypertensive medication (79%), aspirin (74%), statins (55%) and treatment for diabetes (11%) at the time of study entry.

**Table 1:** Baseline patient characteristics (N = 709)

	**Overall**	**Manchester**	**Liverpool**
	**(N = 709)**	**(n = 335)**	**(n = 374)**
Age in y, (mean, range)	66.8 (27-93)	65.1(27-90)	68.4(31-93)
Age ≥ 65, in %	62.2	57.0	66.8
Gender, male %	58.7	57.3	59.9
Smoking status, %			
Never smoker, %	26.4	26.3	26.5
Ex-smoker, %	41.3	41.5	41.2
Current smoker	32.3	32.2	32.4
BMI, mean (median)	27.7(27.1)	27.6(27.1)	27.7(27)
Index event, in %			
Cerebral TIA	43.7	44.5	43.1
Minor stroke	42.3	51	34.5
Retinal TIA	8.9	4.2	13.1
Retinal stroke	5.1	0.3	9.4
Co- morbidity, %			
Previous stroke/TIA	40.1	36.7	43.7
Coronary artery disease	25.7	25.7	25.7
Hypertension	70.2	73.1	67.7
Diabetes mellitus	16.6	15.2	17.9
Medication use, %			
Aspirin	73.8	74.9	72.7
Other antiplatelet drugs	21.3	16.7	25.4
Statins	54.6	55.5	53.7
Angiotensin converting enzyme inhibitor	28.9	30.5	27.5
Angiotensin II receptor blocker	10.3	11.0	9.6
Other anti-hypertensive medication	50.1	50.5	49.7
Anti-anginal therapy	17.9	17.6	18.2

Mean concentrations of CO, PM_10_, NO and NO_2_ were high in Manchester, whereas those of SO_2_ and ozone were higher in Liverpool (Table 2). The daily mean concentration of CO and NO in Manchester was roughly twice that of Liverpool and mean concentration of NO_2_ was 10 μg/m^3^ more than that measured in Liverpool. On the other hand, the concentration of SO_2_ was more than twice as high in Liverpool as in Manchester. The table also shows large daily variations in the distribution of all measured pollutants and temperature during the study period for both regions.

**Table 2:** Descriptive statistics for environmental variables, May 2003 – Jan 2006

	**Manchester**					**Liverpool**				
**Environmental variable**	**Mean**	SD^*^	**Q1**	**Median**	**Q3**	**Mean**	SD^*^	**Q1**	**Median**	**Q3**
CO^†^(mg/m^3^)	0,4	0,2	0,28	0,35	0,43	0,19	0,11	0,15	0,15	0,2
PM_10_^†^(μg/m^3^)	22,6	8,8	16,67	20,33	26	20,57	8,3	15	18,5	23,5
NO^†^(μg/m^3^)	16,9	22,1	7,2	10,2	16,2	8,05	11,49	3,5	5	8
NO_2_^†^(μg/m^3^)	32,5	12,4	23,5	30,5	39,25	21,3	11,13	12,5	18,5	27
SO_2_^†^(μg/m^3^)	2,8	2,8	1	2	4	6,13	3,35	4	5,5	8
Ozone(μg/m^3^)	37,4	16,71	26,67	38	48	47,78	17,5	37	49	60
Temperature(°C)	10,72	5,06	7,02	10,92	14,58	11,25	4,78	7,75	11,42	15,09
Relative humidity, %	79,69	8,36	73,84	80,28	86,17	82,81	8,15	77,61	83,3	89,25

* *SD*: standard deviation; Q1: 25th percentile; Q3: 25th percentile.

† *PM*_10_: mass concentration of particles less than 10 μm in aerodynamic diameter; *CO*: carbon monoxide, *NO*: nitric oxide; *NO*_2_: nitrogen dioxide; SO_2_: sulphur dioxide.

The pairwise correlation coefficients for average daily pollutants and meteorological variables for Manchester are shown in Table 3. Strong correlation was observed between NO and CO (r = 0.89). Similarly, a high correlation between CO and NO_2_ with r = 0.75 was observed. Ozone was negatively correlated with all other pollutants. The largest negative correlation of ozone was with NO_2_ (r = -0.68). Ambient temperature was negatively correlated with all pollutants except ozone. Similar correlation pattern in air pollutants and meteorological variables was observed for data from Liverpool.

**Table 3:** Correlations of mean daily concentrations of pollutants and meteorological parameters, Manchester, May 2003 – Jan 2006

	**CO**	**PM**_**10**_	**NO**	**NO**_**2**_	**SO**_**2**_	**Ozone**	T^*^	**Rh**^**†**^
CO	1							
PM_10_	0.53	1						
NO	0.89	0.49	1					
NO_2_	0.75	0.54	0.77	1				
SO_2_	0.50	0.61	0.49	0.55	1			
Ozone	-0.54	-0.23	-0.59	-0.68	-0.38	1		
T^*^	-0.39	-0.04	-0.42	-0.48	-0.20	0.20	1	
Rh^†^	0.31	-0.09	0.32	0.30	-0.06	-0.50	-0.25	1

*CO*: carbon monoxide; *PM*_10_: mass concentration of particles less than 10 μm in aerodynamic diameter; *NO*: nitric oxide; *NO*_2_: nitrogen dioxide; *SO*_2_: sulphur dioxide;

* Temperature.

† Relative humidity.

### Associations with TIA and minor stroke

The associations between ambient pollutants and the occurrence of TIA and minor stroke are shown in Table 4.

**Table 4:** Associations between ambient particulates and gases and TIA for all study subjects

		**Manchester**		**Liverpool**	
**Lag (days)**	Pollutant^*^	**OR**^**†**^	**95% CI**^**†**^	**OR**	**95% CI**
0	CO	0.95	0.84, 1.07	1.06	0.99, 1.12
0	PM_10_	0.93	0.81, 1.07	0.87	0.76, 0.98
0	NO	0.96	0.90, 1.02	1.04	0.99, 1.09
0	NO_2_	0.94	0.77, 1.14	1.10	0.91, 1.33
0	SO_2_	0.93	0.79, 1.08	0.93	0.79, 1.09
0	O_3_	1.15	0.94, 1.40	0.83	0.68, 1.01
1	CO	0.96	0.85, 1.08	1.02	0.96, 1.09
1	PM_10_	0.93	0.81, 1.06	1.07	0.95, 1.20
1	NO	0.97	0.92, 1.04	1.01	0.96, 1.06
1	NO_2_	1.01	0.84, 1.21	1.06	0.87, 1.28
1	SO_2_	0.95	0.82, 1.09	1.14	0.98, 1.32
1	O_3_	0.90	0.74, 1.10	1.06	0.87, 1.30
2	CO	0.98	0.87, 1.09	1.01	0.95, 1.08
2	PM_10_	0.95	0.84, 1.09	0.94	0.82, 1.06
2	NO	0.97	0.92, 1.03	1.03	0.98, 1.09
2	NO_2_	0.89	0.74, 1.08	0.95	0.79, 1.15
2	SO_2_	0.98	0.85, 1.13	1.03	0.88, 1.21
2	O_3_	1.06	0.87, 1.30	1.09	0.89, 1.33
3	CO	1.08	0.97, 1.19	0.95	0.88, 1.02
3	PM_10_	1.12	0.99, 1.27	0.95	0.83, 1.08
3	NO	1.06	1.01, 1.11	0.93	0.86, 1.00
3	NO_2_	1.12	0.93, 1.35	0.91	0.75, 1.11
3	SO_2_	1.03	0.89, 1.19	0.98	0.83, 1.17
3	O_3_	0.87	0.71, 1.07	1.17	0.95, 1.44

* *CO*: carbon monoxide; *PM*_10_: mass concentration of particles less than 10 μm in aerodynamic diameter; *NO*: nitric oxide; *NO*_2_: nitrogen dioxide; *SO*_2_: sulphur dioxide; *O*_3_: ozone.

† *OR*: odds ratio; *CI*: confidence interval.

For Manchester data, a statistically significant positive association was observed between NO and TIA and minor stroke for lag 3 with estimated OR of 1.06 (95% CI: 1.01-1.11%). Effects of similar or larger magnitude which were not significant were observed for all other pollutants except ozone, for lag 3 in Manchester. For example, an IQR increase in CO was associated with an OR of 1.08 (95% CI: 0.97-1.19), whereas an IQR in PM_10_ was associated with an OR of 1.12 (95% CI: 0.99-1.27). Similarly, marginally non-significant effects were observed with various pollutants at different lags in both cities. In a single pollutant model, no statistically significant positive association was found in Liverpool for any pollutant, apart from a negative effect of PM_10_ at lag 1 and NO for lag 3. All effect estimates at lag 3 for Liverpool data were below unity, except for ozone. A large but non-significant effect of ozone was observed in both regions, with OR of 1.15 (95% CI: 0.94–1.4) for Manchester at lag 0 and 1.17 (95% CI: 0.95–1.44) for Liverpool at lag 3.

### Effect modification

Evidence of effect modification by age, gender and season was observed for both centres (data not shown). In participants above the age of 65 from Manchester, the OR of TIA and minor stroke for an IQR increase of CO and NO at lag 3 were 1.19 (95% CI: 1.03-1.38) and 1.11 (95% CI: 1.03-1.19) respectively when compared to those below 65. Most effect estimates for those below 65 years were close to unity and were non-significant. In addition, an increased risk of TIA and minor stroke in males was observed at lag 3 in association with NO, with an OR of 1.09 (95% CI: 1.01-1.17). Large but non-significant effects of CO, PM_10_ and NO_2_ for lag 3 were observed for Manchester data. Finally, an increased risk of TIA and minor stroke in association with the cold season was observed for both Manchester and Liverpool data.

### Two pollutant model

**Figure 1:**
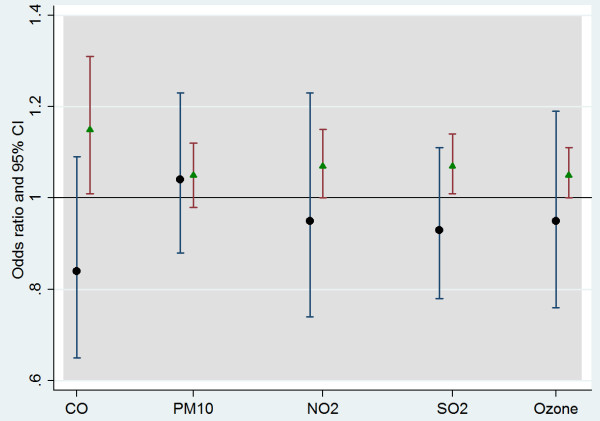
**Adjusted OR and 95% CI in a two pollutant model, lag 3.** The figure shows the result of a two pollutant model analysis on lag 3. The black circles and the squares are odds ratios, vertical lines 95% CI calculated for an IQR of pollutants in consideration. The triangles indicate the estimates for NO, whereas the circles show the estimate for the other pollutant (as specified on the horizontal axis) with which a comparison is being made.

After modelling each pollutant individually, a two-pollutant model was fitted to estimate the independent effect of each pollutant at lag 3 where significant association was observed with NO (Figure 1). The figure reveals that the effect of NO remained stronger when analysed in combination with CO and SO_2._ The effect of NO was borderline significant with NO_2_ and ozone, and non-significant with PM_10_; however, the estimates were above unity and slightly larger than that of PM_10_.

Results obtained from bi-directional referent selection scheme revealed comparable effect estimates to those obtained from the time stratified design (Additional file 1, Table S1).

## Discussion

This study examined the short-term effects of ambient air pollutants on the risk of TIA and minor stroke in a cohort of patients attending TIA services in five UK centres. To date, this is the first study to have specifically investigated the effect of ambient air pollutants on the occurrence of TIA and minor stroke. Its main finding is that NO may be associated with the onset of TIA and minor stroke. A statistically significant association between the occurrence of TIA and minor stroke and exposure to NO with a 3-day lag was observed for the Manchester dataset. In Liverpool, there was no significant positive association between TIA and minor stroke and any of the pollutants, but negative association were observed with PM_10_ and NO at lag 0 and 3 respectively. The study also found effect modification by age, gender and season. Measured pollutants have had more pronounced effects in those above 65 years old, in male and in cold months than in participants below 65 years, female and warmer months respectively.

Reported regression results of the study are based on statistically significant associations, but a statistically non-significant association does not necessarily mean that a relationship does not exist. This study has found several marginally non-significant associations between various pollutants and TIA in both cities, where the lack of significance might be attributed to the small study size in the two centres.

The observed association between NO and TIA and minor stroke could be attributed to chance, due to multiple testing undertaken in the study. We have performed a total of 48 logistic regression analysis and with a conventional 5% significance level, one would expect some of these estimates would achieve this level of ‘significance’ occurred by chance. Thus, the interpretation of the results needs caution and the results need to be corroborated by other studies.

The findings of earlier studies that investigated associations between different subgroups of stroke mortality and morbidity are inconsistent, with some studies reporting increased risk of stroke mortality [11,28-30], and morbidity [2,4-6,31-33], while others found no association [16,18]. These variations may be attributed to the concentration level (and exposure gradient), composition and distribution of the exposure parameter, the degree of exposure misclassification, type of studied pollutant, underlying health status of study population and the method of ascertainment of study outcome.

Although several studies have investigated associations between ambient air pollutants and stroke, fewer have studied these pollutants on TIA [4-6]. A recent US study found an association between same-day and previous day exposure to fine particles and ozone and ischaemic stroke, as well as TIA [5]. A Canadian study found a positive association between same-day exposures to SO_2_ and increased emergency department visits for TIA in the warm season [6]. Similarly, a French study reported a significant association between ozone exposure and risk of TIA at lag of one day [4]. While our study has found a positive association of NO with TIA, none of these three studies have investigated the effect of NO. Furthermore, the effects of NO in our study was observed at lag of 3 days, while the other studies found association on the same day or at lag of one day.

Some studies have found associations between ambient air pollutants and certain stroke subtypes in towns where the concentrations of air pollutants were relatively high. Tsai and others found associations of particles and gases with ischaemic and haemorrhagic stroke when the ambient temperature was above 20°C in Kaohsiung, Taiwan [12]. While most studies have found stronger evidence for the association of ambient air pollutants with ischaemic stroke, a few have reported positive associations of haemorrhagic stroke with ambient PM and gases [12] or total suspended particles [11].

Nitrogen oxide emissions occur largely from motor vehicles in the form of NO, which is a major primary pollutant and precursor of NO_2._ NO typically comprises about 95% of oxides of nitrogen (NO_x_) from a combustion source [34]. Because of the high correlations among measured pollutants in the current study, it is difficult to separate their individual effects; however, NO may be associated with TIA and minor stroke more than the other pollutants as its effect persisted in a two pollutant model. However, we are unable to explain the contradictory negative effect of NO in Liverpool. Perhaps differences in the overall pollution profile and composition might be responsible for the observed effect. The biological mechanism by which NO might be associated with an increased risk of stroke or TIA has not been elucidated so far and there is little information from published literature. Various studies have linked NO_2_ to increased morbidity [18,35], yet related substances like NO are uncommonly investigated in most epidemiological studies. Those few studies that have included NO as an exposure metric have highlighted its positive associations with the respective health outcomes [31,36] as well as a higher correlation with UFP [36] which suggests that NO is an important pollutant to be considered in epidemiological studies.

NO, through a previously unrecognized mechanism, may be responsible for the observed association. NO is endogenously generated and involved in various regulatory and inflammatory functions of the vascular endothelium. It is possible that ambient NO influences bioavailability of endogenous NO. Inhibition of NO and the concomitant changes are known to contribute to essential hypertension, myocardial ischaemia, atherogenesis, thrombosis, insulin resistance and heart failure [37]. A few experimental studies have shown that exposure to nanoparticles and diesel exhaust, which contains significant amounts of NO and UFP, is associated with impairment of vascular function and vasoconstriction, possibly through inhibition of endogenous NO synthesis [38-40]. A recent experimental study which has compared the vasculotoxic responses to various combustion source pollutants has found a significant role of monoxide gases of NO and CO in vascular toxicity [41].

It is possible that NO is a surrogate for other unmeasured pollutants such as ultrafine particles (UFP) [42]. A study in three UK conurbations, including Manchester, has shown a strong correlation between UFP and NO_x_ (r = 0.85) and CO (r = 0.78) [43]. A Finnish study reported a significant association between UFP and reduced peak expiratory flow rate (PEFR), a measure of lung function [36]. In that study, UFP was highly correlated with NO (r = 0.77) and the effect of UFP on PEFR diminished in a two-pollutant model when paired with traffic-related gaseous pollutants CO, NO and NO_2_[36].

One strength of our study is that the diagnosis of TIA or minor stroke was confirmed by a consultant stroke physician or neurologist independent of the study. Most previous studies have used hospital admission or discharge diagnostic code data to establish diagnosis which carries a significant risk of misclassification [44,45]. The study has a number of limitations. Firstly, participants were recruited at first presentation to secondary care services which in some cases was as late as 6 weeks and this might lead to recall bias. This was standard clinical care in the UK at the time (2003-7) but may also have introduced selection bias as higher-risk patients may have had a subsequent stroke prior to presenting to TIA services. Participants included in this study may therefore likely to represent lower- risk cases of TIA and minor stroke. There could be a differential effect of pollution on low and high risk cases which would affect the generalizability of the results. Secondly, the exposure data were obtained from fixed central site monitors which do not necessarily reflect personal exposure, especially for traffic related gaseous pollutants that are not spatially homogenous like NO and NO_2_ and this may have led to exposure misclassification. Additionally, as most individuals spend most of their time indoors, use of exposure data from outdoor central monitors will introduce exposure measurement error. However, such error is likely to be non-differential and would be expected to underestimate the observed effects [46]. Study numbers are also relatively low, particularly as for geographic reasons they had to be divided into two populations.

## Conclusions

This study has shown a modest positive association between ambient NO and the occurrence of TIA and minor stroke, in Manchester, which was modified by age, gender and season. Given the large number of multiple comparisons performed, the finding may have occurred by chance and additional epidemiological research with a larger sample size in a different study population is required to confirm this finding.

## Abbreviations

BMI: Body mass index; CI: Confidence interval; CO: Carbon monoxide; CV: Cardiovascular; IHD: Ischaemic Heart Disease; IQR: Inter quartile range; NO: Nitric oxide; NO_2_: Nitrogen dioxide; NO_x_: Oxides of nitrogen; NORTHSTAR: The North West of England Transient Ischaemic Attack and Minor Stroke study; O_3_: Ozone; OR: Odds ratio; PM_10_: Mass of particles with aerodynamic diameter less than 10 μm; SD: Standard deviation; SO_2_: Sulphur dioxide; TIA: Transient ischaemic attack; UFP: Ultrafine particles.

## Competing interests

All authors declare that they have no competing interests.

## Authors’ contributions

GBB designed the study, collected air pollution data, planned and performed the statistical analyses and drafted the manuscript. CJS, PJT and RA contributed to the study design, interpretation of the results and preparation of the manuscript. CJS and PJT established the TIA and minor stroke cohort and provided the cohort data. AAH contributed to the interpretation of the results and preparation of the manuscript. All authors read and approved the final manuscript.

## Supplementary Material

Additional file 1 **Table S1.** Associations between ambient particulates and gases and TIA for all study subjects: sensitivity analysis in a bilateral case-crossover strategy. Click here for file
