# Supplementary material for: Short-term effects of ambient particulates and gaseous pollutants on the incidence of transient ischaemic attack and minor stroke: a case-crossover study
Source: Environ Health. 2012 Oct 15;11:77. doi: 10.1186/1476-069X-11-77 (PMC3533825; doi:10.1186/1476-069X-11-77)
Supplement: Additional file 1 — Table S1. Associations between ambient particulates and gases and TIA for all study subjects: sensitivity analysis in a bilateral case-crossover strategy. [file 1476-069X-11-77-S1.pdf]

Supplemental Material to:

Short-term effects of ambient particulates and gaseous pollutants on the incidence of transient ischaemic attack and minor stroke: a case- crossover study

G. Bero Bedada, C.J. Smith, P.J. Tyrrell, A.A. Hirst, R. Agius

Table s1. Associations between ambient particulates and gases and TIA for all study subjects: sensitivity analysis in a bilateral case-crossover strategy

		Manchester		Liverpool	
Lag (days)	Pollutant*	OR†	95% CI†	OR	95% CI
0	CO	0.93	0.81 , 1.05	1.04	0.95 , 1.13
0	PM_{10}	0.93	0.82 , 1.02	0.90	0.77 , 1.02
0	NO	0.96	0.87 , 1.02	1.03	0.97 , 1.09
0	NO_2	0.94	0.78 , 1.11	1.10	0.92 , 1.28
0	SO_2	0.95	0.86 , 1.04	0.94	0.82 , 1.06
0	O_3	1.14	0.96 , 1.33	0.85	0.66 , 1.03
1	CO	0.97	0.87 , 1.08	1.01	0.93 , 1.09
1	PM_{10}	0.94	0.80 , 1.08	1.07	0.93 , 1.21
1	NO	0.96	0.90 , 1.01	1.02	0.94 , 1.09
1	NO_2	0.99	0.86 , 1.12	1.06	0.85 , 1.28
1	SO_2	0.97	0.84 , 1.09	1.13	0.94 , 1.32
1	O_3	0.87	0.71 , 1.03	1.06	0.85 , 1.27
2	CO	1.00	0.90 , 1.10	0.98	0.92 , 1.04
2	PM_{10}	0.93	0.83 , 1.03	0.96	0.85 , 1.06
2	NO	0.97	0.89 , 1.04	1.02	0.96 , 1.09
2	NO_2	0.91	0.78 , 1.04	0.97	0.81 , 1.13
2	SO_2	0.99	0.86 , 1.11	1.05	0.90 , 1.20
2	O_3	1.06	0.89 , 1.23	1.08	0.86 , 1.30
3	CO	1.06	0.96 , 1.16	0.95	0.87 , 1.03
3	PM_{10}	1.10	0.95 , 1.25	0.97	0.85 , 1.10
3	NO	1.05	1.00 , 1.10	0.95	0.87 , 1.03
3	NO_2	1.11	0.95 , 1.26	0.93	0.73 , 1.12

3	SO_2	1.01	0.91 , 1.11	0.98	0.84 , 1.12
3	O_3	0.90	0.75 , 1.04	1.16	0.92 , 1.40

* CO: carbon monoxide; PM_{10}: mass concentration of particles less than 10 μm in aerodynamic diameter; NO: nitric oxide; NO_2: nitrogen dioxide; SO_2: sulphur dioxide; O_3: ozone

† OR: odds ratio; CI: confidence interval
